# Autocrine parathyroid hormone-like hormone promotes intrahepatic cholangiocarcinoma cell proliferation via increased ERK/JNK-ATF2-cyclinD1 signaling

**DOI:** 10.1186/s12967-017-1342-1

**Published:** 2017-11-25

**Authors:** Jing Tang, Yan Liao, Shuying He, Jie Shi, Liang Peng, Xiaoping Xu, Fang Xie, Na Diao, Jinlan Huang, Qian Xie, Chuang Lin, Xiaoying Luo, Kaili Liao, Juanjuan Ma, Jingyi Li, Daichao Zhou, Zhijun Li, Jun Xu, Chao Zhong, Guozhen Wang, Lan Bai

**Affiliations:** 1 Guangdong Provincial Key Laboratory of Gastroenterology, Department of Gastroenterology, Nanfang Hospital, Southern Medical University, No. 1838, Guangzhou Avenue North, Baiyun District, Guangzhou, Guangdong China; 2 Laboratory Medicine Center, Nanfang Hospital, Southern Medical University, Guangzhou, Guangdong China; 3 Department of Gastroenterology, Dali Bai Autonomous Prefecture People’s Hospital, Dali, Yunnan China; 4 Department of Pathology, Nanfang Hospital, Southern Medical University, Guangzhou, Guangdong China

**Keywords:** Parathyroid hormone-like hormone, Activating transcription factor-2, Proliferation, Intrahepatic cholangiocarcinoma

## Abstract

**Background and aims:**

Intrahepatic cholangiocarcinoma (ICC) is an aggressive tumor with a high fatality rate. It was recently found that parathyroid hormone-like hormone (PTHLH) was frequently overexpressed in ICC compared with non-tumor tissue. This study aimed to elucidate the underlying mechanisms of PTHLH in ICC development.

**Methods:**

The CCK-8 assay, colony formation assays, flow cytometry and a xenograft model were used to examine the role of PTHLH in ICC cells proliferation. Immunohistochemistry (IHC) and western blot assays were used to detect target proteins. Luciferase reporter, chromatin immunoprecipitation (ChIP) and DNA pull-down assays were used to verify the transcription regulation of activating transcription factor-2 (ATF2).

**Results:**

PTHLH was significantly upregulated in ICC compared with adjacent and normal tissues. Upregulation of PTHLH indicated a poor pathological differentiation and intrahepatic metastasis. Functional study demonstrated that PTHLH silencing markedly suppressed ICC cells growth, while specific overexpression of PTHLH has the opposite effect. Mechanistically, secreted PTHLH could promote ICC cell growth by activating extracellular signal-related kinase (ERK) and c-Jun N-terminal kinase (JNK) signaling pathways, and subsequently upregulated ATF2 and cyclinD1 expression. Further study found that the promoter activity of PTHLH were negatively regulated by ATF2, indicating that a negative feedback loop exists.

**Conclusions:**

Our findings demonstrated that the ICC-secreted PTHLH plays a characteristic growth-promoting role through activating the canonical ERK/JNK-ATF2-cyclinD1 signaling pathways in ICC development. We identified a negative feedback loop formed by ATF2 and PTHLH. In this study, we explored the therapeutic implication for ICC patients.

**Electronic supplementary material:**

The online version of this article (10.1186/s12967-017-1342-1) contains supplementary material, which is available to authorized users.

## Background

Intrahepatic cholangiocarcinoma arises from epithelial cells lining the bile duct and occurs proximally at the second degree bile ducts within the liver. The condition is commonly associated with cirrhosis, viral hepatitis B and C [1]. ICC displays a feature of rapid progression and a poor outcome, and its global disease incidence has been rapidly increasing [2]. Radical resection and curative liver transplantation are preferred surgical treatments for ICC, however, the patients with vascular and lymph nodes metastases are not eligible for surgical therapy. Although the chemotherapy regimen of gemcitabine and cisplatin and locoregional therapies are additional options for inoperable ICC patients, the 5-year survival rates are very low. An enhanced understanding of the biology pathological progress and the interaction with tumor microenvironment of ICC is needed to improve patient survival.

PTHLH, also referred to parathyroid hormone-related protein (PTHrP), has emerged as an important cytokine with diverse cell functions, including growth, survival, migration, and differentiation [3]. Tumor-derived PTHLH participates in the bone metastatic processes of breast cancer via an intracrine fashion [4]. In addition, PTHLH supports colorectal cancer cell to form distant lung metastatic processes via inducing caspase-independent death in human lung vasculature endothelial cells [5]. A previous report demonstrated that PTHLH produced by proliferating bile duct epithelial cells and may interact with growth factors and hormones to form complex loops that promotes proliferation [6]. Growing evidences indicate that PTHLH-producing cholangiocarcinoma (CHO) patients suffer from humoral hypercalcemia of malignancy [7–9], but litter is known regarding PTHLH’s effect on ICC cells growth. MAP kinase pathways are involved in the process through the PTHLH-induced activation of PTH1R to activate downstream effectors [10–12]. ATF2, as a downstream effector of MAPK in response to cytokines, is phosphorylated on Thr69 and/or Thr71 by either JNK or p38 [13, 14], and is also activated by the ERK1/2 pathway in two step [13]. Several observations support that ATF2 regulates cell cycle progression via controlling the transcriptional output of several key genes, including CyclinD1, CyclinA and RB1 [15–18]. However, no studies have documented a role for the PTHLH-MAPK-ATF2-CyclinD1 signaling axis in the regulation of ICC cells growth. This study aims to elucidate the role and clinical significance of the PTHLH-MAPK-ATF2-CyclinD1 axis in ICC cell cycle progression.

## Methods

### Patients, tissue samples and microarrays

59 ICC samples and paired non-tumor tissues and 10 normal tissues were obtained from the Department of Hepatobiliary Surgery, NanFang Hospital, Southern Medical University between 2014 and 2016. All patients signed informed consent for therapy and subsequent tissue studies, which were approved by the NanFang Hospital Institutional Review Board. The ICC tissue microarrays, which contained 100 cases, and the extrahepatic cholangiocarcinoma (ECC) tissue microarrays, which contained 27 cases were purchased from Shanghai Outdo Biotech Inc. (Shanghai, China). All tumors were defined as a primary tumor arising from the bile ducts and diagnosed as adenocarcinomas. Tumor stage was defined according to the seventh edition of American Joint Committee on Cancer/International Union against Cancer (AJCC/UICC). All specimens were used for routine pathological processing with comparable clinicopathological features, and complete follow-up data were obtained.

### Western blot, real-time PCR analysis, immunohistochemistry and immunofluorescence

RNA and protein lysate extraction, cDNA synthesis, final real-time PCR and western blots were performed according to general protocols. ICC cells were processed for immunofluorescence (IF) using target antibodies with optimized conditions. In addition, human samples and ICC microarrays were subjected to IHC staining to evaluate the expression of relative proteins.

### Cell counting kit-8 assay, colony formation assays, cell cycle analysis, cell migration and invasion assay

Cell counting kit-8 assay, colony formation assays, cell cycle analysis, cell migration and invasion assay and Annexin V apoptosis assay were performed according to general protocols and can be found in Additional file 1.

### Dual-luciferase reporter gene assay

To determine the effect of ATF2 on PTHLH transcription, RBE cells were transfected with pGL3 as vehicle control, pGL3-PTHLH or pGL3-MUT-PTHLH using Lipofectamine 3000. Firefly and Renilla luciferase activities were measured separately on a fluorescence spectrophotometer (FlOUstar omega, BMG Labtech, Germany) in triplicate according to the manufacturer’s instructions for the dual-luciferase reporter assay kit (Promega). The relative transcriptional activity was normalized by the corresponding vehicle control value.

### Chromatin immunoprecipitation (ChIP) assay

Genomic DNA prepared from RBE cells transfected with shControl was crosslinked with 1% formaldehyde and fragmented into 500 ± 100-bp fragments by sonication. Soluble chromatin was then incubated overnight with anti-ATF2 antibodies. Finally, the immunoprecipitated DNA fragments were amplified and quantified using real-time PCR using the following PCR primers specific to the human PTHLH promoter region.

### Establishment of a subcutaneous tumor xenograft

RBE cells (shCtrl or shPTHLHx) (1 × 10^7^) were injected subcutaneously into the groins of BALB/c nude mice (6 weeks old, male, n = 5 for each group). Tumor growth was monitored at 2 or 3-day intervals. When the mice were sacrificed after 25 days, tumor weight and size were measured, and the tumor was fixed for additional experimental use.

### Statistical analyses

Different statistical analysis methods were used to compare different groups or different categories of data. Extended details regarding materials and methods can be found in Additional file 1.

## Results

### PTHLH is highly expressed in human CHO tissues specimens and ICC cell lines

**Fig. 1:**
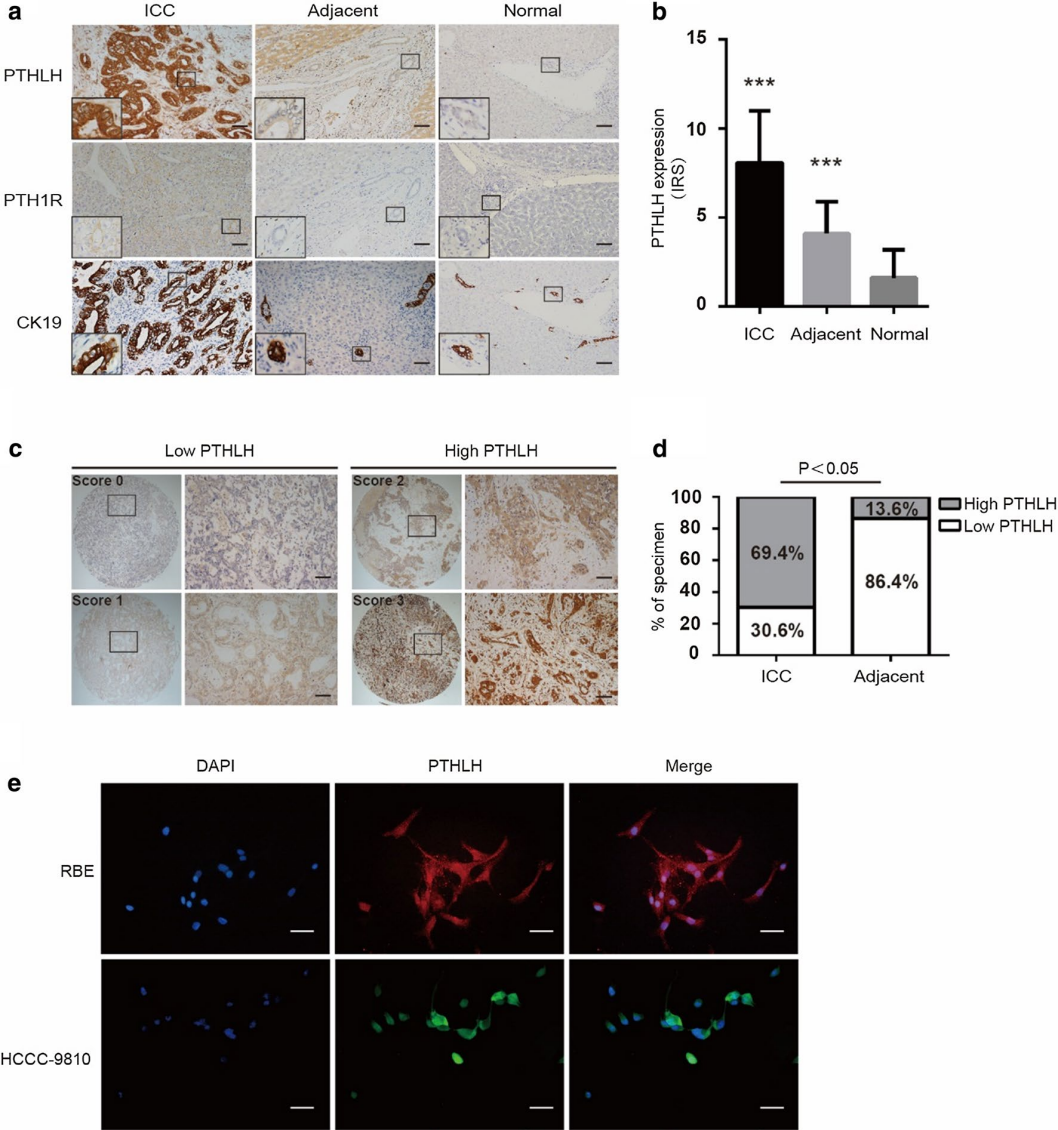
PTHLH is highly expressed in ICC tissues and cells. **a** Representative images of PTHLH, PTH1R and CK19 protein expression in ICC tumor tissues, paired adjacent tissues and normal tissues as assessed by immunohistochemistry (DAB staining, scale bar, 50 μm). **b** PTHLH protein expression was significantly increased in primary ICC tumors as compared with their adjacent and normal tissues (***p < 0.01). **c**, **d** Scores indicate PTHLH levels in ICC (DAB staining, scale bar, 50 μm). The scores were calculated by intensity and the percentage of stained cells as described in Additional file 1 (DAB staining, scale bar, 50 μm). **e** Immunofluorescence location. PTHLH was expressed in both nuclear and cytoplasm in ICC cell lines (HCCC-9810 and RBE) by immunofluorescence and fluorescent microscopy (scale bar, 50 μm)

To identify the potential role of PTHLH in CHO, we evaluated 59 ICC samples and paired non-tumor tissues from NanFang Hospital. We also screened an additional 10 samples of normal liver tissues for comparison. IHC analyses of ICC tumor regions revealed strong staining of PTHLH compared with that in adjacent regions in the same patients (Fig. 1a, top panel and b). Immunostaining of PTHLP protein was located in the cytoplasm and nucleus of ICC cells. We also observed weak staining in the bile duct of adjacent and normal tissue samples (Fig. 1a, top panel). In addition, we detected the expression of PTH1R, a specific receptor for PTHLH, in membranes of ICC cells (Fig. 1a, middle panel). Cytokeratin19 (CK) staining revealed the presence of adenocarcinoma cells and biliary epithelial cells (Fig. 1a, bottom panel). In addition, to further confirm the expression of PTHLH in CHO, we screened ICC microarrays that contained 100 cases and the ECC microarrays that contained 27 cases (Fig. 1c and Additional file 1: Figure S1). Our results were consistent with the conclusion above that PTHLH was highly expressed in CHO cells. In addition, PTHLH protein expression was examined in ICC cell lines by IF microscopy (Fig. 1e). Microscopy analysis detected cytosolic and nucleus expression of PTHLH in RBE and HCCC-9810 cells, which is consistent with previous observations.

### Overexpression PTHLH is positively correlated with poor pathological differentiation in ICC patients

To investigate the clinical significance of PTHLH upregulation in CHO, we further analyzed the relationship between clinicopathological features and PTHLH expression levels in CHO cases. These patients were divided into high- (score, 2–3) or low- (score, 0–1) PTHLH expression groups according to the immunostaining scores (Fig. 1c, d). Scoring was conducted according to the ratio and intensity of positive-staining cells: 0–5% scored 0; 6–35% scored 1; 36–70% scored 2; more than 70% scored 3. The final score was designated as low or high expression group as follows: score 0–1, low expression, score 2–3, high expression. A high expression of PTHLH was positively correlated with poor pathological differentiation (p < 0.05) (Table 1). These findings indicate that PTHLH expression might contribute to ICC progression and be a potential therapeutic target of this disease.

**Table 1:** Correlation of the ICC clinicopathological features and TNM staging with PTHLH expression (related to Fig. 1)

Variable	N	PTHLH expression in ICC (n = 108) (%)		χ2	*p* value
		High (75)	Low (33)		
Age (years)					
≤50	25	18 (72.0)	7 (28.0)	0.100	NS
>50	83	57 (68.7)	26 (31.3)		
Gender					
Female	43	33 (76.7)	10 (23.3)	1.794	NS
Male	65	42 (64.6)	23 (35.4)		
Pathological differentiation					
I	8	5 (62.5)	3 (37.5)	7.236	*0.027*
II	53	31 (58.5)	22 (41.5)		
III	47	39 (83.0)	8 (17.0)		
Tumor size (cm)					
≥5	71	49 (69.0)	22 (31.0)	0.018	NS
<5	37	26 (70.3)	11 (29.7)		
Intrahepatic metastasis					
Yes	44	24 (54.5)	20 (45.5)	7.768	*0.005*
No	64	51 (79.7)	13 (20.3)		
Distant metastasis					
Yes	5	4 (80.0)	1 (10.0)	0.275	NS
No	103	71 (68.9)	32 (31.1)		
TNM					
I–II	72	24 (33.3)	48 (66.7)	1.544	NS
III–IV	36	9 (25.0)	27 (75.0)		

Italic values indicate significance of *p* value (*p* < 0.05)

*NS* not significant between any groups

Note: ICC patients were divided into PTHLH ‘High’ group and ‘Low’ group

Abbreviations: ICC, Intrahepatic cholangiocarcinoma; Differences among variables were assessed by χ^2^ or Fisher’s exact χ^2^ test

### PTHLH promotes ICC cells growth

**Fig. 2:**
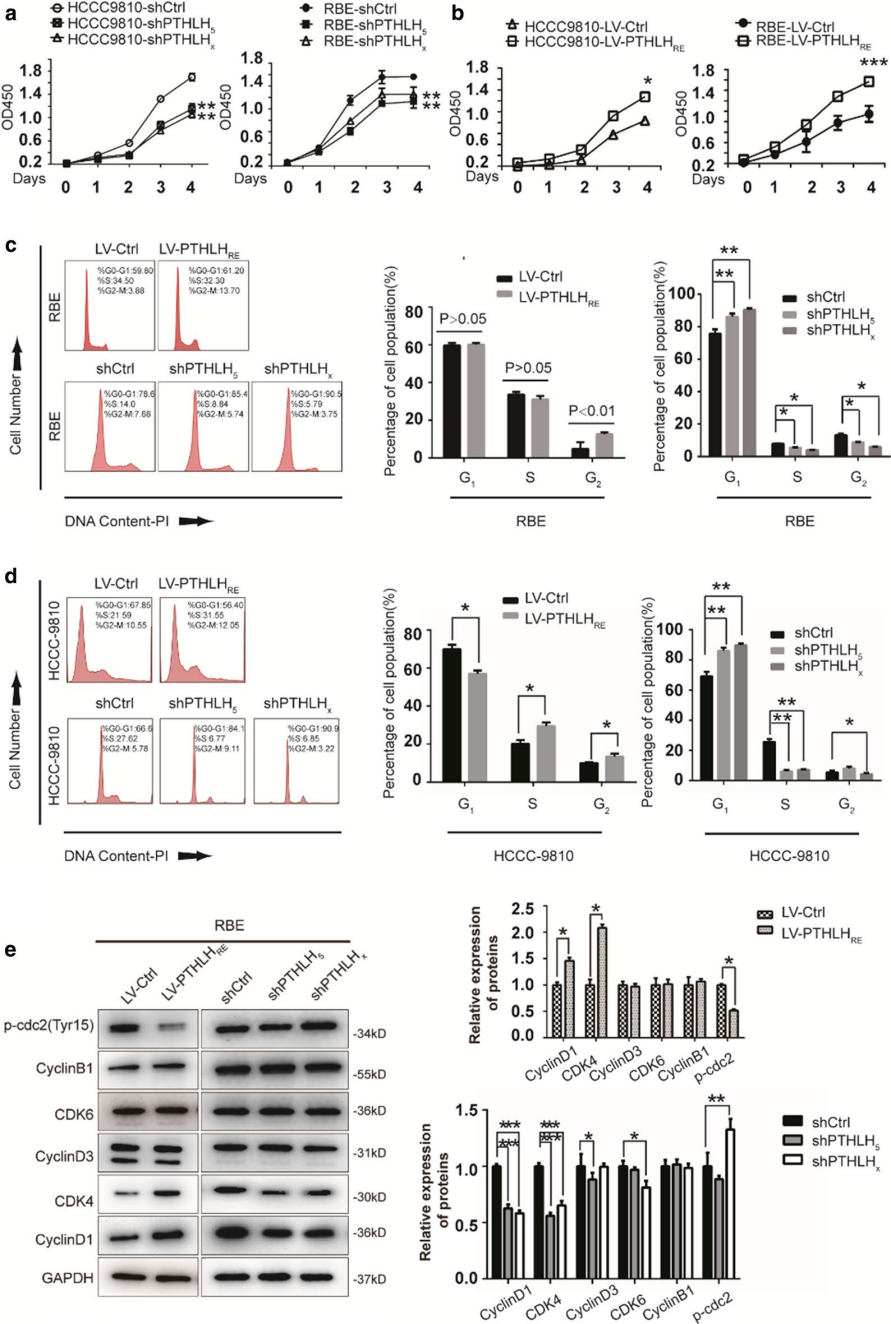
PTHLH promotes ICC cell growth via altering the cell cycle. **a** Depletion of PTHLH suppresses ICC cell growth (**p < 0.01). **b** PTHLH re-expression accelerates cell growth in ICC cells. Cell proliferation was examined using the CCK-8 assay in ICC cells with the stable re-expression of PTHLH (*p < 0.05, ***p < 0.001). **c** The distribution of cell cycle phases in RBE cells. The values are the mean ± SD (*< 0.05, **< 0.01). **d** The distribution of cell cycle phases in HCCC-9810 cells. Cell population sizes averaged from three independent experiments with standard deviations (*< 0.05, **< 0.01). **e** Protein expression of CyclinB1, p-cdc2, cell-cycle-related proteins in the G_1_ phase was determined by western blot (*< 0.05, **< 0.01, ***< 0.001)

The above data suggested that PTHLH may play a critical role in ICC progression. To address whether PTHLH affects cell proliferation, we first investigated endogenous PTHLH levels in tow ICC cell lines. We observed they both have PTHLH endogenous expression (Additional file 1: Figure S2A). We then generated two PTHLH-specific shRNAs to silence the endogenous PTHLH expression in ICC cells. shPTHLHx, which induced the most significant knock-down (KD) effect, was used for vivo study. We stably depleted PTHLH in RBE and HCCC-9810 cells. The relative expression of PTHLH in RBE and HCCC-9810 cells was confirmed by qPCR and western blot (Additional file 1: Figure S2B, C). PTHLH depletion significantly decreased ICC cell proliferation (Fig. 2a and Additional file 1: Figure S3). To evaluate the effects of PTHLH re-expression on tumor growth in vitro, we knock-down endogenous PTHLH and then reintroduced lentivirus-mediated vector (LV-Ctrl) and PTHLH using lentivirus-mediated PTHLH_GFP_ (LV-PTHLH_RE_) to examine whether the re-expression of PTHLH could rescue the retarded proliferation (Additional file 1: Figure S2D). Compared with the control, incubation with PTHLH-specific shRNA resulted in elongated morphology cells and less confluent cell growth. When exposed to lentivirus-mediated PTHLH_GFP_, cell growth returned to normal (Additional file 1: Figure S3). Furthermore, we observed that PTHLH secretion was upregulated upon the reintroduction of PTHLH, indicating an autocrine function of PTHLH (Additional file 1: Figure S2E). Similarly, PTHLH re-expression increased LV-PTHLH_RE_ ICC cell proliferation (Fig. 2b and Additional file 1: Figure S3).

### PTHLH alters the cell cycle distribution

Cell cycle distribution was analyzed by flow cytometry analysis to determine whether PTHLH enhances cell growth and promotes tumorigenesis via an alteration of the cell cycle. After 12 h of serum starvation for synchronization, the cell population in the G_2_/M phase is significantly increased upon PTHLH re-expression, whereas the G_0_/G_1_ and S phase cell population remained more constant in RBE cells (Fig. 2c top panel). In contrast, the reverse effect was observed when PTHLH was depleted. shPTHLH arrested RBE cells at the G_0_/G_1_ phase, and the proportion of cells in the S and G_2_/M phase decreased (Fig. 2c bottom panel). These results demonstrate that PTHLH re-expression facilitates the S to G_2_/M phase transition. However, PTHLH deletion blocks the cell cycle by inhibiting the G_0_/G_1_ to S phase transition. The similar results were obtained from another ICC cell line, HCCC-9810 (Fig. 2d). To further explore the molecular basis of PTHLH-enhanced tumour development, we investigated the roles of PTHLH on metastasis using in vitro migration and Matrigel invasion assays. The results indicate that PTHLH facilitating RBE cells migration not invasion (Additional file 1: Figure S4A). We also found no significant differences between migration and invasion of HCCC-9810 cells (Additional file 1: Figure S4B). We quantitatively investigated the effect of PTHLH on apoptosis by flow cytometry after staining with Annexin V and 7-amino-actinomycin. The results indicate that ICC cell apoptosis is not regulated by PTHLH (Additional file 1: Figure S4C).

### PTHLH regulates the expression of genes controlling the cell cycle

The observed differences in the cell cycle distribution were due to the different expression levels of key cell cycle proteins. We noted an increased accumulation of G_2_/M-phase cells upon PTHLH re-expression compared with that in LV-Ctrl cells. We observed that p-cdc2 protein levels decreased significantly, whereas CyclinB1 levels remains constant when PTHLH was re-expressed (Fig. 2e, left panel). These results indicate that PTHLH re-expression promotes RBE cells mitosis via downregulating p-cdc2 expression. We also detected G_0_/G_1_ phase-related proteins (CDK4/CyclinD1 and CDK6/Cyclin D3). CDK4/CyclinD1 protein levels increased slightly, whereas CDK6 and Cyclin D3 levels remained constant (Fig. 2e, left panel). Given that the cell cycle was altered by PTHLH depletion, we focused our attention on key proteins (CDK4/CyclinD1 and CDK6/Cyclin D3) during the G_0_/G_1_ phase. Western blot analysis indicated that CyclinD1 and CDK4 protein levels decreased dramatically when PTHLH expression was deleted in RBE cells (Fig. 2e, right panel). In contrast, Cyclin D3 and CDK6 expression remained constant. These data suggested that PTHLH regulates the expression of cell cycle-related proteins.

### Loss of PTHLH expression suppresses tumorigenesis in vivo

**Fig. 3:**
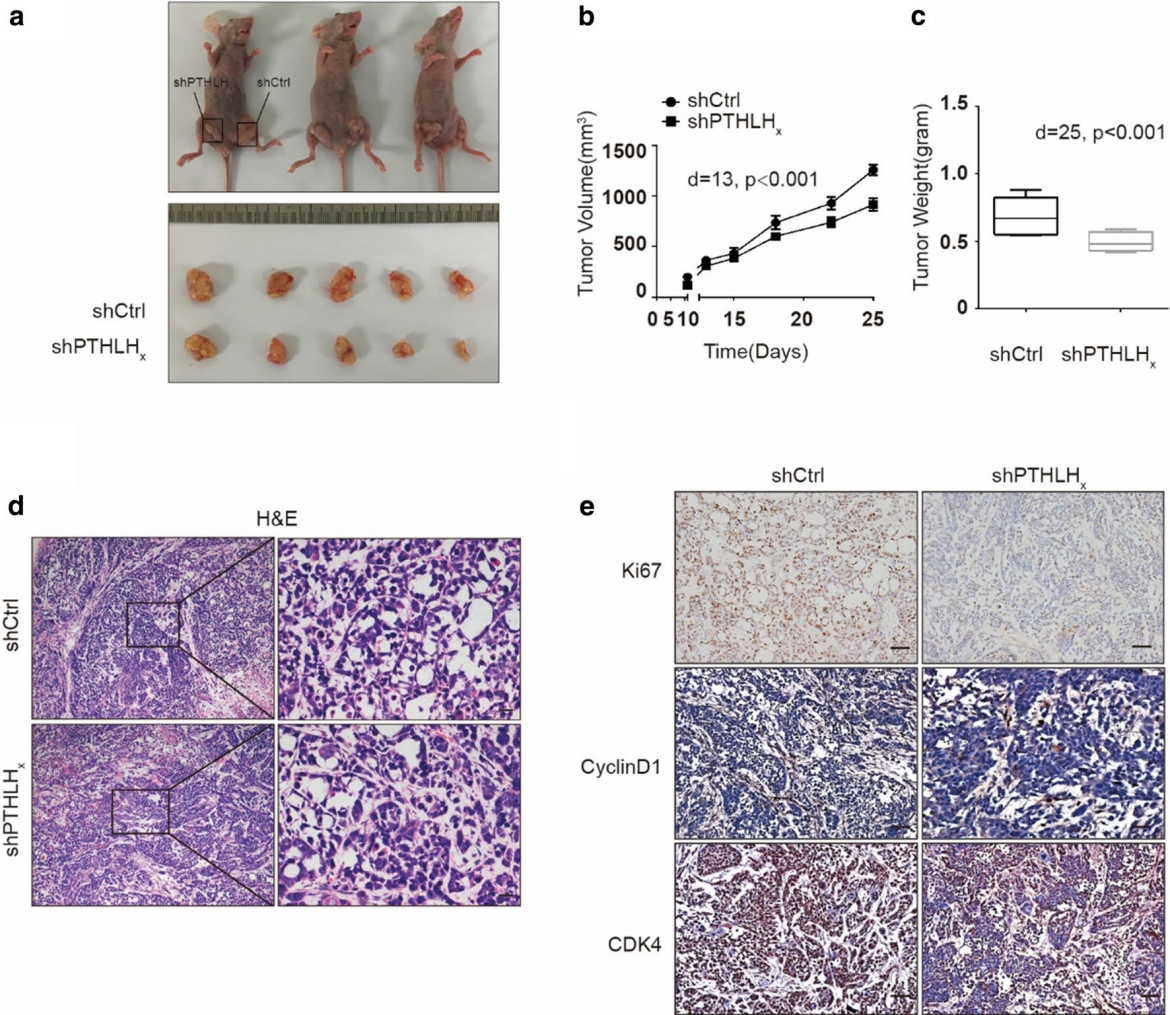
Loss of PTHLH significantly suppressed cell proliferation. **a**–**c** PTHLH deletion suppresses tumor growth. Five nude mice were injected subcutaneously with 1 × 10^7^ cells/mouse for each of the indicated stable cell lines. The results are presented as isolated tumors (**a**), tumor sizes (**b**), and tumor weights (**c**). **d** H&E staining of xenograft tumor from nude mice injected with shCtrl or shPTHLH_x_ cells (scale bar, 20 μm). **e** Representative images of Ki67, CyclinD1, and CDK4 protein expression in xenograft tumors from nude mice as assessed by immunohistochemistry (DAB staining, scale bar, 50 μm)

To investigate whether PTHLH deletion suppresses tumorigenesis in vivo, PTHLH-KD RBE cells (shPTHLHx) were implanted subcutaneously into the right inguen, and vector cells (shCtrl) were implanted into the left inguen of nude mice (n = 5) (Fig. 3a). Tumor growth was monitored as described in Additional file 1. Consistent with the cell proliferation assay in vitro, tumor growth was significantly decreased in mouse xenografts with shPTHLHx compared with that of shCtrl (Fig. 3b, c). Consistently, the nuclear expression of Ki-67, Cyclin D1 and CDK4 proteins was significantly increased in the shCtrl-RBE tumors compared with that in the shPTHLHx-RBE tumors (Fig. 3e), which is consistent with the in vitro study using western blot (Fig. 2e). These results collectively suggest that PTHLH promotes ICC cell proliferation.

### PTHLH altered cell cycle genes via activating phosphorylated ATF2 through JNK/ERK1/2 signaling pathways

**Fig. 4:**
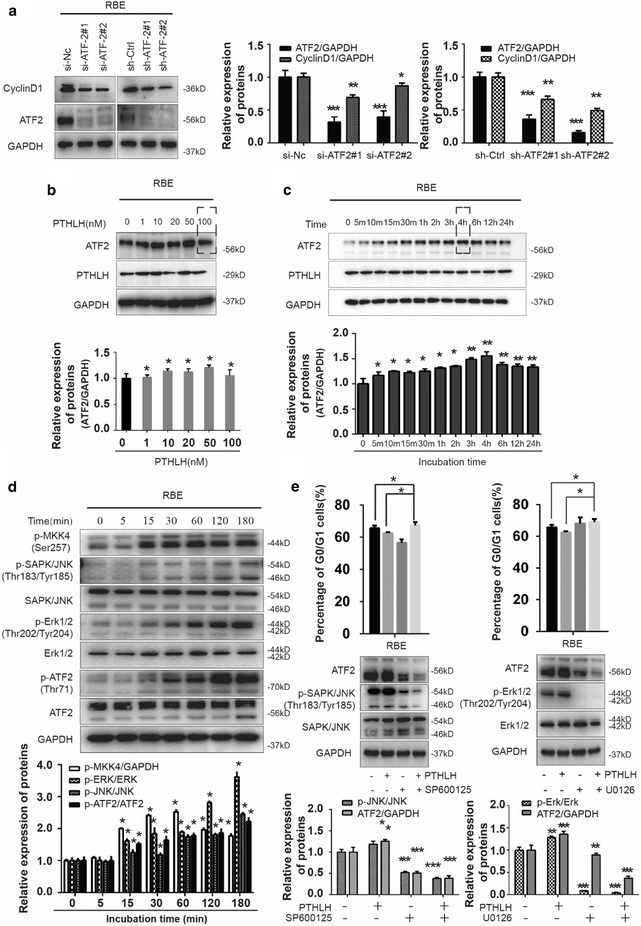
Upstream kinase for ATF2 phosphorylation and expression. **a** ATF2 regulates the expression of CyclinD1. Western blot indicating the protein level of CyclinD1 in RBE cells where ATF-2 was stably or transiently depleted (*< 0.05, **< 0.01, ***< 0.001). **b** The cells were treated with 0, 1, 10, 20, 50, and 100 nM PTHLH for 4 h, and western blot was performed for ATF2, PTHLH and GAPDH (*< 0.05). **c** The cells were treated with 20 nM PTHLH for the indicated times, and western blot was performed for ATF2, PTHLH and GAPDH (*< 0.05, **< 0.01). **d** Western blot of the expression of ATF2, JNK and ERK1/2 protein and phosphorylation of JNK (Thr183/Thr185), ERK1/2 (T202/Y204), MKK4 (S257) and ATF-2 (Thr71) in the RBE cells (*< 0.05). **e** Cells were pre-treated with U0126 (10 μM) or SP600125 (20 μM) and then treated with 20 nM PTHLH for 4 h. Cell distribution was determined by flow cytometry (*< 0.05), and the expression of related proteins was determined by western blot (**< 0.01, ***< 0.001)

Accumulating studies highlight PTHLH as a cellular cytokine with actions involved in both cell growth and differentiation [3]. Previous reports supported that the PTHLH can trigger the MAPK signaling cascade by binding with PTH1R, which interacts with the MAPK scaffolding protein β-arrestin2 and G-protein [10–12]. As a downstream effector of MAPK, ATF2 regulates cell cycle progression through the transcriptional control of CyclinD1 (Fig. 4a) [18]. We hypothesized that PTHLH can increase ATF2 transcriptional activity by activating ERK1/2 and JNK signaling cascades. As shown in (Fig. 4b, c), we treated RBE cells with PTHLH (1–34) recombination fragment and assessed the ATF2 expression. PTHLH (1–34) induced a time- and dose-responsive increase of in ATF2 protein expression. In contrast, the effect of PTHLH was attenuated at 100 nM (Fig. 4b, dotted line). These results indicated that PTHLH exhibits dose-dependent biphasic effects on ICC cell dynamics. We also found endogenous PTHLH re-expression upregulated ATF2 protein expression (Additional file 1: Figure S5). In response to PTHLH stimulation, PTHLH/PTH1R signaling triggers JNK and ERK1/2 signaling pathways (Fig. 4d). Therefore, pharmacological approaches were used to confirm that ATF2 transcriptional activity is regulated by PTHLH. When RBE cells were pretreated with an MEK1/2 inhibitor (U0126) or JNK1/2 inhibitor (SP600125) for 1 h followed by PTHLH (1–34) treatment for 4 h, we observed that the MEK1/2 and JNK1/2 inhibitor abrogated the PTHLH-induced phosphorylation of ATF2 (p-ATF2) (Fig. 4e), suggesting that p-ATF2 induction involves the PTHLH-JNK/ERK1/2 signaling cascade. Interestingly, we further found that the MEK inhibitor U0126 and the JNK inhibitor SP600125 inhibited RBE cell growth, arresting cells at the G_0_/G_1_ phase (Fig. 4e). These results suggesting that the inhibition of JNK/ERK1/2 attenuated PTHLH-induced ICC growth.

### ATF2 negatively regulate PTHLH expression

**Fig. 5:**
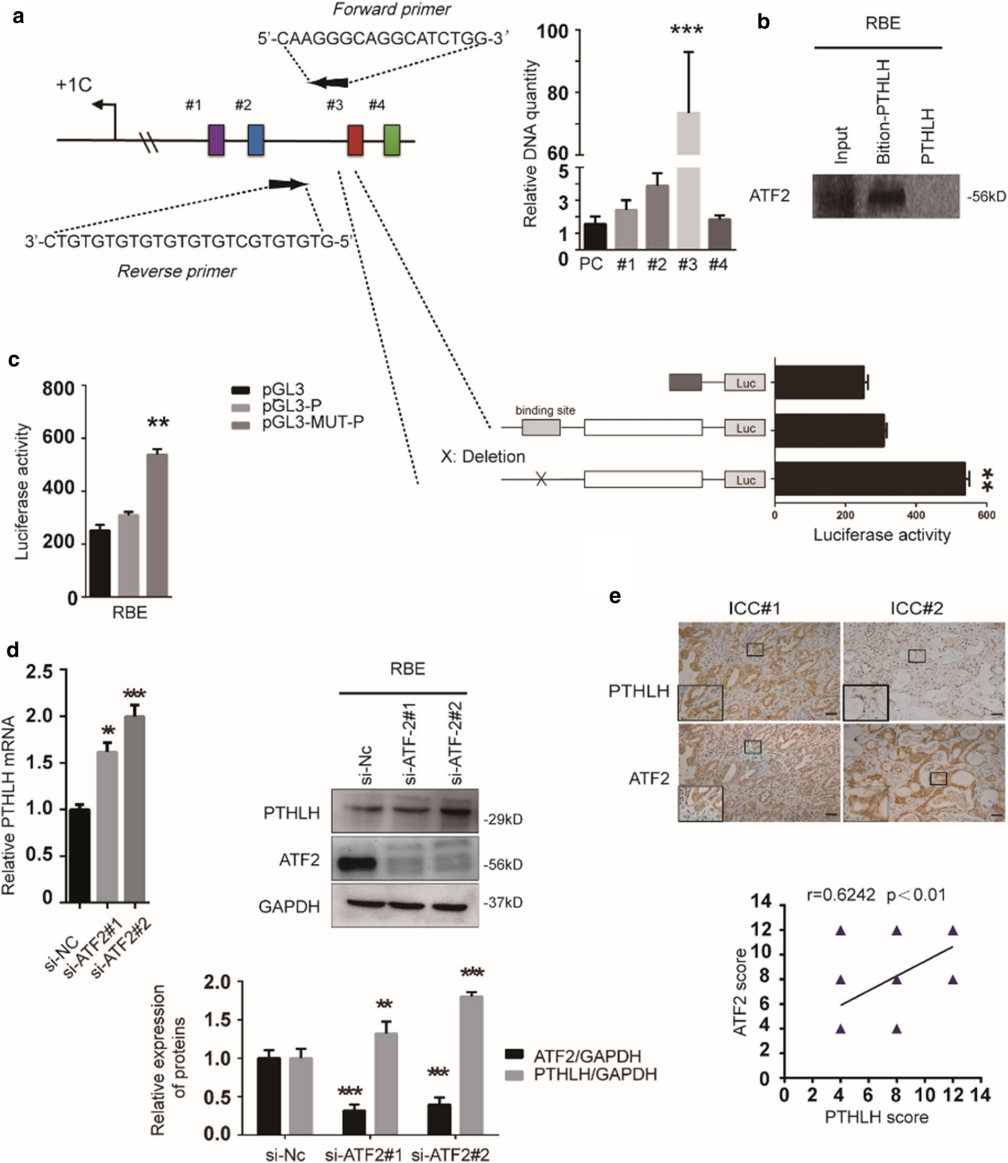
Mapping the ATF2 response element on the PTHLH promoter. **a** Structure of the PTHLH promoter. Putative ATF2 response elements and fragments of the promoter that were cloned are depicted (colorful boxes, region “#1”–“#4”). Mutation of ATF2 element in the chromatin immunoprecipitation assay. Immunoprecipitated DNA was used as the template in PCR with primers corresponding to the proximal region of the PTHLH promoter (***p < 0.001). PC primers were used as control. **b** DNA pull-down assay. An equal amount of cell lysate was pulled down with biotinylated PTHLH DNA probe followed by immunoblotting with anti-ATF2 antibody. **c** Mutation of a PTHLH element at site #3 inhibits reporter activity. PTHLH was mutated, and the relative luciferase activity of the WT and mutant construct was assessed in RBC cells. The results are presented as the mean ± SD. The data were standardized to β-galactosidase (**p < 0.01). **d** An equal amount of cell lysate from RBE cells transfected with ATF2 small interfering RNA plasmid (si-ATF2), control plasmids (si-NC) or control was subjected to qPCR and immunoblotting with PTHLH or ATF-2 antibody, GAPDH reveals equal loading. **e** Stains of the same cohorts of ICC sections for analysis of the related expression of PTHLH and ATF2

ATF2 is a bZIP transcription factors which has an ability to bind to the CRE consensus. According to previous reports, the *pthlh* gene contains a CRE element within its promoter region. Interestingly, we also found that ATF2 might interact with PTHLH promoter elements in bioinformatics prediction methods (Additional file 1: Figure S5). Next, we mapped the ATF2 response element(s) on the PTHLH promoter. Analysis of the proximal region revealed the presence of ATF2 target sequences at positions − 2210 to − 2243 (Site #3) (Fig. 5a). Further support for the role of ATF2 in the regulation of PTHLH transcription was provided by ChIP analysis. Sheared chromatin was immunoprecipitated with antibodies to ATF-2 (or control IgG) followed by the PCR amplification of PTHLH promoter sequences. Immunoprecipitation of ATF2 enabled the amplification of PTHLH promoter sequences, demonstrating the in vivo binding of ATF2 to the PTHLH promoter. DNA pull-down assays demonstrated ATF2 binding to the PTHLH promoter region (Fig. 5b). Consistent with this finding, a mutation within this site attenuated the basal level of reporter activity and the binding of ATF2 (Fig. 5c), confirming that ATF2 regulates PTHLH transcription via binding its response element at the Site #3. Our studies also indicated that PTHLH expression was regulated by si-ATF2, as confirmed by qPCR and western blot (Fig. 5d). To further confirm the correlation between PTHLH and ATF2 expression in ICC, we detected ATF2 expression using the same samples (Fig. 5e). Further statistical analysis revealed that the ATF2 expression correlated with PTHLH expression in the tissue samples (r = 0.624, p < 0.05), suggesting a potentially complicated regulatory mechanism between PTHLH and ATF2.

## Discussion

ICC is one of the most lethal epithelial cancers united by poor diagnoses and adverse outcomes. The condition frequently arises in the presence of chronic injury and inflammation. Previous literatures documenting that ICC is commonly associated with cirrhosis, viral hepatitis B and C, and metabolic abnormalities [19–22]. The molecular pathogenesis of ICC proliferation and metastasis, as the main cause of ICC-related mortality, but the mechanisms remain obscure. Our findings demonstrate that PTHLH knockdown in ICC cells suppressed tumor growth, while re-expression of PTHLH has the opposite effect, highlighting the role of PTHLH as a critical oncoprotein in ICC progression.

PTHLH/PTH1R signaling is aberrantly induced or activated in different cancer types and is associated with poor prognosis [5, 23–25]. In the present study, we found that ICC cells produced PTHLH ligands that respond via the expression of cognate receptors PTH1R, resulting in the continuous activation of downstream signaling pathways. Extensive evidence suggest that PTHLH is viewed as a cellular cytokine, particularly in epithelial cancer cells, that exhibits an autocrine or paracrine role in both cell growth and differentiation [26, 27]. In our previous study, we found that overexpression of the PTHLH (LV-PTHLH), which transfects lentivirus-mediated PTHLH_GFP_ without deleting endogenous PTHLH expression in ICC cells, may enter into the non-proliferative cells (data not show). In contrast, LV-PTHLH_RE_ ICC cells promoted cell growth. Moreover, endogenous PTHLH sustains the activation of MAPK signaling pathways, and this effect was more pronounced after the addition of a PTHLH recombination peptide. In contrast, the effect of PTHLH on activation was attenuated at a higher concentration (100 nM) compared with 50 nM (Fig. 5b, dotted portion). These results clearly indicated that PTHLH may function as a tumor cell growth promoter within a certain concentration range. When the range is exceeding, PTHLH becomes saturated and suppresses cells proliferation. In our study, we also found re-expression PTHLH promotes RBE cells migration and specific overexpression PTHLH associates with intrahepatic metastasis in ICC patients. All results indicated a potential ability of facilitating tumor invasiveness. The data would suggest that PTHLH may potentially transform ICC cells into an aggressive form of the disease. And this also indicates that PTHLH influences ICC cells growth and differentiation with a low signal expression.

**Fig. 6:**
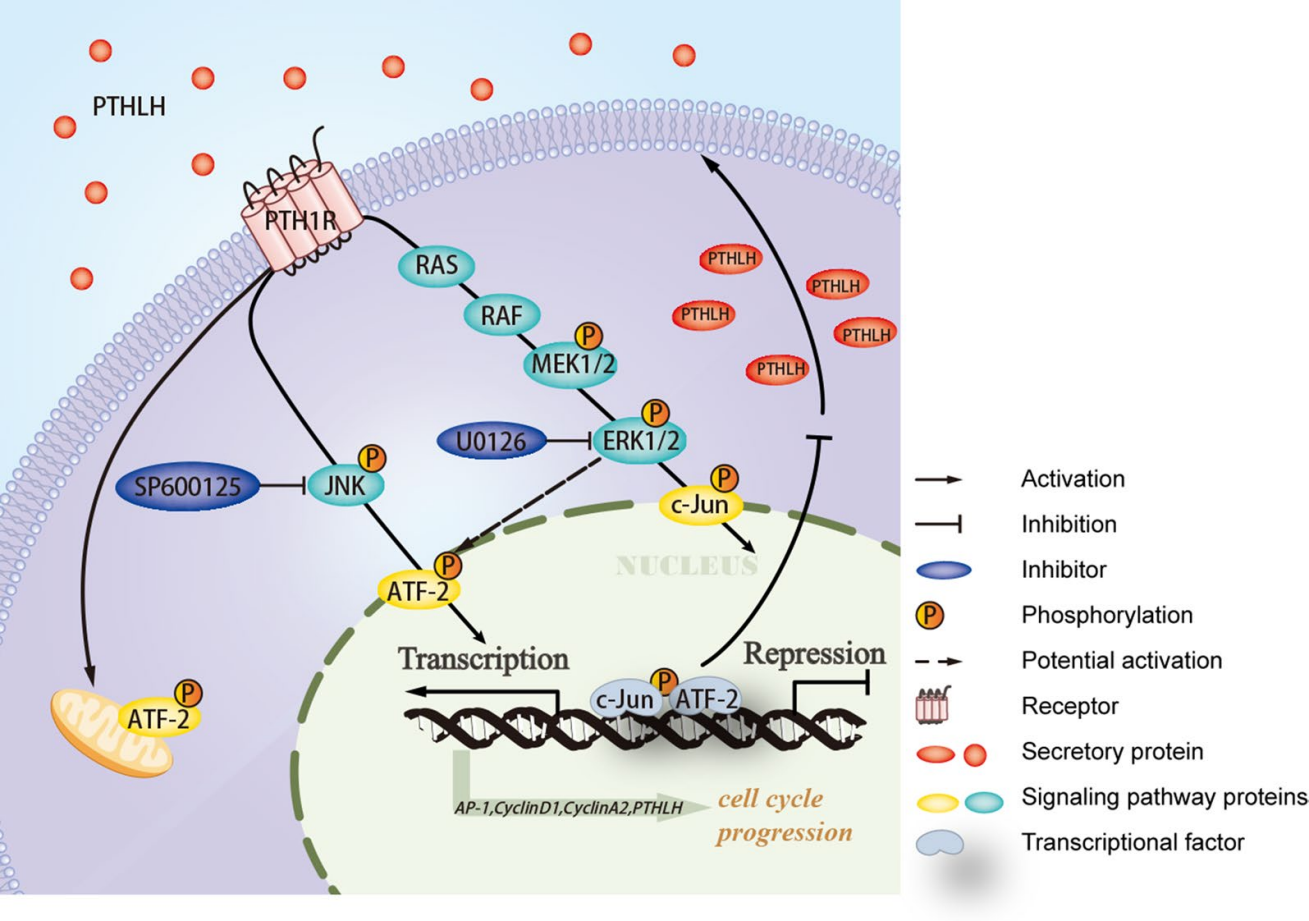
A model of the PTHLH/PTH1R-ATF2 negative-feedback loop. PTHLH/PTH1R signaling activated the expression and transcriptional activity of ATF2 via the ERK1/2 and JNK signaling pathways to promoted ICC cell growth, and ATF2 positively regulated CyclinD1 expression and negatively regulated PTHLH expression. Treatment with ERK1/2 and JNK signaling pathway inhibitors abolished ATF2 transcriptional activity

To investigate the effect of ICC secreted PTHLH on cancer cell growth, we established an in-vitro PTHLH secretion system. In our present work, we found that PTHLH regulated cell growth by altering the cell cycle. Cell cycle dysregulation is a major feature of tumorigenesis, which occurs by shortening the G_1_ phase or activating CDKs may favor tumor development [28]. Herein, we demonstrate that PTHLH controlled cell cycle progression. Secreted PTHLH protein act on target cells by binding to its specific cell surface receptor: PTH1R. The potential molecular mechanisms could be explained by the finding that the PTHLH protein activates the JNK/ERK1/2-ATF2 axis via interacting with the MAPK scaffolding protein β-arrestin2, or triggering an early G protein-dependent pathway meditated by PKA and PKC [10] leading to cell cycle proliferation. Furthermore, we provided evidence suggesting that PTHLH upregulates ATF2 phosphorylation via activating the ERK1/2 and JNK signaling pathways, which transcriptionally upregulate CyclinD1 expression. When ERK1/2 and JNK are pharmacologically inhibited in ICC cells (i.e., via U0126 and SP600125), which blocked PTHLH-induced activation of ERK1/2 and JNK signal pathways, and transcriptional activity of ATF2 (Fig. 6). Cyclin D1 is frequently deregulated in cancer and is a biomarker of cancer phenotypes and disease progression [29]. Previous findings suggested that the deregulation of CyclinD1 expression and CDK4 activation directly lead to some cancer hallmarks by inducing proliferation [30–32]. We observed that reduced CyclinD1 expression and CDK4 inactivation directly inhibited proliferation, which is consistent with previous reports. Consistently, our present work demonstrated that PTHLH re-expression accelerated the G_2_ to M phase transition, which is similar to the effects of numerous other oncogenes. Among the genes functioning during the G_2_ and M phase transition, we observed that the p-cdc2 levels were rapidly reduced upon PTHLH re-expression. Several experimental findings indicate that cdc2 is one of the master regulators of mitosis that controls the centrosome cycle in complex with A- or B-type cyclins [33]. Previous reports have suggested that cdc2 activity upon mitosis entry depends on p-cdc2 levels [34]. However, the reduction of cdc2 activity primarily drives the exit from mitosis [33]. Our present study indicates that PTHLH promote mitosis in ICC cells via downregulating p-cdc2 expression. Interestingly, we observed a paradoxical phenomenon that PTHLH overexpression without knocking-down endogenous secretion arrests ICC cells in the G_1_ phase and decreases CyclinD1 expression (data not show). We hypothesized that ostensibly paradoxical responses between PTHLH deletion and overexpression in cultured ICC cells appear to facilitate a compromise between maximal mitogenic stimulation and the avoidance of antiproliferative defenses.

Another interesting finding of this study is the negative regulated role of ATF2 in RBE cells (Fig. 6). ATF2 is an important transcription factor that can facilitate malignant proliferation. In our previous study, we found that ATF2 promoted growth of ICC cells and was correlated with a poor prognosis for ICC patients. In vitro evidence indicated that the upregulation of ATF2 phosphorylation and activity promotes cancer progression via facilitating cell proliferation-related gene expression. In our study, we demonstrated that PTHLH can promote oncogenic functions of ATF2 by activating ERK/JNK pathways. And a previous report revealed PTHLH can activate PKC pathway [35]. Eric Lau [36] previously reported that PKC_ε_ promotes oncogenic functions of ATF2 in the nucleus while blocking its apoptotic function at mitochondria. Thus, we believe that PTHLH promotes nuclear translocation and transcriptional function in the oncogenic functions of ATF2. Interestingly, another novel finding of our study is the negative role of ATF2 in PTHLH production in REB cells. And we also found autocrine activation of the PTHLH promoter by c-Jun (data not show). Collectively, these findings indicated that ATF2 limits PTHLH transcriptional output to maintain specific concentration by forming a homodimer or a heterodimer with JUN. It is possible that cancer cells may have negative feedback loops that are essential for survival.

## Conclusions

In summary, we report that ICC-secreted PTHLH acts in an autocrine manner in intrahepatic cholangiocarcinoma progression by activating the canonical ERK/JNK signaling pathway. However, our findings focus on only PTHLH-mediated ICC cell proliferation, not provide new insight into the ICC metastasis. Despite the importance of PTHLH tumorigenic role, our knowledge of the PTH1R that mediate changes in the tumor progression and interaction with PTHLH in ICC is still limited. Based on our findings, further investigation for interfering with PTH1R, which mediate signaling in cancer cells, may serve as effective treatment approaches to ICC patients. And we will improve the mechanisms of PTHLH/PTH1R-mediated ICC progression and involve the interaction of the transcription factors CREB and AP-1 (c-JUN, c-FOS and ATF2) in ICC development.

## Additional file

**Additional file 1.** Additional Figures.

## Abbreviations

ICC: intrahepatic cholangiocarcinoma
PTHLH: parathyroid hormone-like hormone
PTHrP: parathyroid hormone-related protein
IHC: immunohistochemistry
ChIP: chromatin immunoprecipitation
ATF2: activating transcription factor-2
PTH1R: PTH type 1 receptor
ECC: extrahepatic cholangiocarcinoma
IF: immunofluorescence
PI: propidium iodide
CK19: cytokeratin19
CHO: cholangiocarcinoma
KD: knockdown
