# Supplementary material for: Autocrine parathyroid hormone-like hormone promotes intrahepatic cholangiocarcinoma cell proliferation via increased ERK/JNK-ATF2-cyclinD1 signaling
Source: J Transl Med. 2017 Nov 25;15:238. doi: 10.1186/s12967-017-1342-1 (PMC5702246; doi:10.1186/s12967-017-1342-1)
Supplement: Supplementary file 1 — Additional file 1. Additional Figures. [file 12967_2017_1342_MOESM1_ESM.docx]

**Autocrine Parathyroid Hormone-like Hormone Promotes Intrahepatic Cholangiocarcinoma Cell Proliferation via increased ERK/JNK-ATF2-CyclinD1 signaling**

Jing Tang, Yan Liao, Shuying He, Jie Shi, Liang Peng, Xiaoping Xu, Fang Xie, Na Diao, Jinlan Huang, Qian Xie, Chuang Lin, Xiaoying Luo, Kaili Liao, Juanjuan Ma, Jingyi Li, Daichao Zhou, Zhijun Li, Jun Xu, Chao Zhong, Guozhen Wang, LanBai

**Table of contents**

**Additional materials and methods**

**Additional Figure S1**

**Additional Figure S2**

**Additional Figure S3**

**Additional Figure S4**

**Additional Figure S5**

**Additional Figure S6**

**Additional Figure S7**

**References**

**Additional materials and methods**

***Cell culture and establishment of stable cell Lines***

Immortalized human ICC cell lines (RBE, HCCC-9810), and H293T cells were purchased from the Cell Bank of the Chinese Academy of Sciences. RBE and HCCC-9810 were cultured in RPMI-1640 (Invitrogen, Carlsbad, CA) supplemented with 10% (v/v) FBS (Gibco, US). H293T cells were cultured in Dulbecco’s modified Eagle medium (DMEM, Invitrogen) containing 10% (v/v) FBS (Gibco) at 37^0^C in a humidified atmosphere of 5% CO2 condition.

***Primary antibodies for western blot analysis, imunohistochemistry and immunofluorescence***

The following primary antibodies were used in Western blot analysis:

GAPDH(16A00406, ZSGB-BIO, Wuhan, China), ATF2(A2155, ABclonal, Wuhan, China), PTHLH(ab115488, Abcam, Cambridge, MA), JNK (9252, Cell Signaling Technology, US), ERK1/2 (4695, CST), P-ATF2 (AP0020, ABclonal), P-ERK1/2 (4370, CST), P-JNK (4668, CST), P-MKK4 (4514, CST), p-cdc2(4539, CST), Cyclin B1(12231, CST), Cyclin D1(2978, CST), Cyclin D3(2936, CST), CDK6(3136, CST), CDK4(12790, CST)

Primary antibodies for imunohistochemistry and immunofluorescence:

PTHLH (677939, Abnova, Taipei, Taiwan), PTH1R (A1744, ABclonal), ATF2 (A2155, ABclonal), Cytokeratin19 (ZM-0074, ZSGB-BIO, Peking, China), PTHLH (ab115488, Abcam), Ki67 (AM0241, Ascendbio, Guangzhou, China), Cyclin D1 (AM0081, Ascendbio), CDK4 (AR0560, Ascendbio).

Primary antibodies for chromatin immunoprecipitation and DNA pull down assay:

ATF2 (ab32061, Abcam)

PTH-related protein (1-34) amide (human, mouse, rat) (H-5494, Bachem, US)，MEK1/2 inhibitor (U0126) (Beyotime, Shanghai, China), JNK1/2 inhibitor (SP600125) (Beyotime, Shanghai, China) for Western blot.

***siRNA transfection***

Small interfering RNAs duplexes for ATF2 were produced by Ribobio (Guangzhou, China). Transfection steps were following the manufacture’s protocols.

ATF2#1: (F) 5’- ACGGCGACGATTCTTCCTT -3’

ATF2#2: (F) 5’- CCATCTGATCGCAGAAATC -3’

***Lentivirus production and cell transduction***

Virus packaging was performed in 293T cells after cotransfection of pGLV3-H1-GFP+Puro Vector or pGLV5-EF-1a-GFP+Puro Vector (GenePharma) using Lipofectamine 3000 (Invitrogen). Viruses were harvested 48 and 72 h after transfection, and virus titers were determined. Target cells (1×10^5^), including HCCC-9810 and RBE cells, were infected with 1×10^6^ recombinant lentivirus-transducing units in the presence of 5μg/ml polybrene (GenePharma).

LV3-NC：(F) 5’- TTCTCCGAACGTGTCACGT-3’

shPTHLH_5_：(F) 5’- GATCGCAGAAATCCACACA-3’

shPTHLH_X_：(F) 5’-GGGCAGATACCTAACTCAGGA-3’

***RT-PCR analysis***

Total RNA extracted by using Trizol reagent (Takara, Japan) and then reversely transcribed into cDNA through PrimeScript™ RT reagent Kit (Takara). Real-time PCR analyses were performed with SYBR *Premix Ex Taq*™ II (Takara) on a ROCHE LightCycle 480 QPCR (Swit) system according to the protocol. Primer sequences used are as follows:

PTHLH: (F) 5’-ATTTACGGCGACGATTCTTCC-3’ and

(R) 5’-GCTTGGAGTTAGGGGACACC-3’.

ATF2: (F) 5’-GCACAGCCCACATCAGCTATT-3’ and

(R) 5’-GGTGCCTGGGTGATTACAGT-3’.

GAPDH: (F) 5’-AATGAAGGGGTCATTGATGG-3’ and

(R) 5’-AAGGTGAAGGTCGGAGTCAA-3’.

***Immunofluorescence***

Cultured ICC cells (5 × 10^4^cells) were fixed with 4% paraformaldehyde, permeabilized with 0.1% Triton X-100 in PBS, and blocked with 3% BSA in PBS. Expression of PTHLH was detected using anti-PHTLH primary antibody and visualized with Alexa Fluor 488 and Alexa Fluor 555 -conjugated secondary antibodies (Invitrogen). Nuclei were counterstained with 4’, 6-diamidino-2-phenylindole (DAPI) staining solution (Beyotime, Shanghai, China). All images were viewed using an Olympus XB-51 fluorescence inverted microscope (Olympus, Tokyo, Japan).

***Immunohistochemistry***

Immunostaining was performed using indicated primary antibodies overnight at 4℃. After washing, sections were incubated with IgG/HRP-conjugated secondary antibodies (PV-6001, PV-6002, ZSGB-BIO). The immunostaining was examined with an Olympus BX51 microscope (Olympus). Scoring was conducted according to the ratio and intensity of positive-staining cells: 0-5% scored 0; 6-35% scored 1; 36-70% scored 2; more than 70% scored 3. The final score was designated as low or high expression group as follows: score 0-1, low expression, score 2-3, high expression. These scores were determined independently by two senior pathologists. The scoring by the pathologists was done in a blinded manner.

***Western blot***

Protein lysate extraction of indicated cells were prepared using RIPA lysis buffer containing 1x protease cocktail inhibitor (Sigma). Samples of protein (15–30μg) were loaded onto 10%-12% SDS-polyacrylamide gels for electrophoresis and then separated proteins were transferred to PVDF membranes (Millipore, Billerica, MA). Expression of each protein was detected using indicated primary antibody overnight at 4℃. After incubation with HRP-conjugated secondary antibodies, membranes were developed using ECL (Beyotime, Shanghai, China). The image acquisition of blots was per- formed using a Gene5 image acquisition system (Syngene, Frederick, MD).

***Chromatin immunoprecipitation***

A total of 108 of cells from RBE cells, respectively, were 1% formaldehyde-fixed, lysed and sonicated for 6 × 30 s in a Bioruptor sonicator (Diagenode, Denville, NJ, USA). Supernatants were precleared with protein A/G agarose beads (Roche, Indianapolis, IN, USA). Chromatin fragments were immune-precipitated by using an anti-ATF2 affinity-purified rabbit polyclonal antibody or normal rabbit polyclonal antibody (negative control) and purified using QIAquick PCR purification columns (Qiagen, Hilden, Germany) followed by 40 cycles of PCR amplification. The PCR primer sequences of PTHLH are the following:

Region1: (F) 5’- GATGGAGATTACTCAGTTATGTTAGGAACTAG - 3’

(R) 5’-TAGAAATTCTCCTCAATATATACCCAGGAC -3’

Region2: (F) 5’- CACTGGGGATGGGAAGATG - 3’

(R) 5’-TAGAAATTCTGCTTAGGCCTTGCT -3’

Region3: (F) 5’- CAAGGGCAGGCATCTGG - 3’

(R) 5’- CTGTGTGTGTGTGTGTGTGTGTG -3’

Region4: (F) 5’- TGGGCTGCTGCCAGGA - 3’

(R) 5’- TGGCTGGCCAAGTTTTTGG -3’

***DNA pull down assay***

Cells were lysed by RIPA lysisi buffer containing 1×protease and phosphatase inhibitors for the preparation of nuclear exact. Nuclear extracts were precleared with Streptavidin-agarose resin (Thermo) for 1 hour, then the precleared supernatant was incubated with 1μg biotinylated double-stranded oligonucleotide PTHLH-site: Biotin-5’-GCTGCCAGGAGGGCC-3’ and 5’-CAATGTGAGATGAGAAGGTCTTTAGG-3’ on ice for 4 hours. DNA-bound protein was collected by incubation with streptavidin-agarose resin on a shaker to prevent precipitation overnight at 4°C. The resin bound complex was washed with 1ml Wash Buffer II（50mM Tris-Cl pH7.0, 1mM EDTA，100mM KCl，01%TritonX-100, 5%Glycerol，1mM DTT） by centrifugation at 2000×g for 1-2 minutes and removal of the supernatant for at least five times. After the resin-bound complex denaturation in SDS-PAGE sample buffer, the released antigen were loaded onto the 4-20% SDS-PAGE gel and identified by Western blotting with specific antibodies.

***ICC specimens collection and CHO microarrays***

59 ICC patients were enrolled in this study, who underwent a surgical resection at NanFang Hospital, Southern Medical University (Guangzhou, China) during the period 2014–2016. ICC diagnosis was based on NCCN Clinical Practice Guidelines in Oncology: Hepatobiliary Cancers (Version 2.2016). All samples were confirmed to be adenocarcinomas according to histopathological diagnosis. Tumor differentiation was defined according to the Edmondson grading system. The TNM staging of tumors was determined according to the seventh edition of American Joint Committee on Cancer/International Union against Cancer (AJCC/UICC). Normal cystic duct tissues, used as the control, were obtained from the 10 chronic calculous cholecystitis in NanFang Hospital, Southern Medical University, between 2014 and 2016. The ICC microarrays contained 100 cases and the ECC microarrays contained 27 cases were purchased from Shanghai Outdo Biotech Inc.

***Animal studies***

Male BALB/c athymic nude mice (6 weeks old，male, n=5 for each group) were obtained from the experimental animal centre of Southern Medical University and housed under standard conditions and cared for according to the institutional guidelines for animal care. All of the animal experiments were approved by the Institutional Animal Care and Use Committee (IACUC) of Southern Medical University. To establish ICC mice model, 1×10^7^cells in 150 μl of phosphate-buffered saline were subcutaneously injected into the groins of nude mice. Tumor growth was monitored at two or three-day intervals. When the mice were sacrificed after 25 days, tumor weight and volume were measured and fixed for Hematoxylin and Eosin (H&E) and IHC staining. Xenograft volumes were evaluated by caliper measurements of two perpendicular diameters and calculated individually as formula: Volume = a × b2/2 (a represent length and b represent width).

***Cell counting kit-8 assay***

Cells (5×10^3^/well) were cultured in 96 well plates. After 24 h of treatment, 10 μl of CCK-8 reagent was added to each plate according to the manufacturer’s protocol ([MultiSciences](http://www.liankebio.com/brand-MultiSciences.html), China) for 1-4 h following solubilization, the orange color was detected by spectrophotometer at 450 nm.

***Soft agar colony formation assay***

Transfected ICC cells suspended in 2×RPMI-1640 (20% FBS), were mixed with equal volume of 0.7% agar and seeded into pre-coated 6-well dish at a density of 4×10^4^ cells per well. After 3w, colonies were counted using Image J software (NIH Image).

***Cell cycle analysis***

Cell cycle distribution was determined by measuring the content of nuclear DNA with propidium iodide (PI) staining. In brief, ICC cells cultured in six-well cell culture cluster dishes (2-5×10^5^ cells per well) were washed twice with PBS and maintained in serum-deprived medium for 12 h to synchronize cells at the G_0_ phase. The cells were treated and used for additional experimental procedures. The cells were collected by trypsinization and washed twice with ice-cold PBS. The collected cells were fixed in 70% ethanol at 4°C overnight. The cells were incubated with 100 μg/ml RNase A for 50 minutes at 37°C and then stained with 0.5ml of 100μg/ml PI solution (KeyGEN BioTECH, Jiangsu, China). The stained cells were measured using a flow cytometer (CellQuestTM Pro). The percentage of cells in the G_0_/G_1_, S, and G_2_/M phases of the cell cycle were determined using FlowJo v10.2 software. All experiments were performed in triplicate.

***Cell migration and invasion assay***

Cell migration and invasion assays were performed as standard. Briefly, 500μl cell suspension containing a total of 2.5×10^4^ cells (migration) and 5×10^4^cells (invasion) per well were seeded in the top of chamber of 24-well Transwell chambers (Corning) according to the manufacturer's instructions (BD Biosciences). After 12 (migration) or 24 (invasion) hours of incubation, cells from the top chambers acrossed 8-μm porous membranes toward media with 10% FBS placed in the bottom chamber. The non-migrated cells were subsequently removed from the upper side of the chamber with a cotton swab and the migrated cells were fixed with 4% paraformaldehyde (PFA) and stained with 0.5% crystal violet solution. The average number of migrated cells was determined by counting from 5 to 10 high power fields (HPFs) under the microscope.

***Annexin V apoptosis assay***

Apoptosis was assessed by flow cytometry after staining with Annexin V (FITC-conjugated) (BD Biosciences, Erembodegem, Belgium) and 7-amino-actinomycin (7-AAD) (BD Biosciences). Cell populations were counted as viable (Annexin V-negative, 7-AAD-negative), early apoptotic (Annexin V-positive, 7-AAD-negative), and late apoptotic (Annexin V-positive, 7-AAD-positive), and necrotic cells (Annexin V-negative, 7-AAD-positive). The experiments were performed in triplicate for three times independently.

***Statistical analysis***

Statistical significance of differences between groups was assessed using the GraphPad Prism6 software. Student's t-test or one-way ANOVA was applied to determine the significance between groups. Statistical analyses between different treatments, in different cell cohorts or at different time points were performed using two-way ANOVA with the Bonferroni's correction. The statistical correlation between the clinical parameters of CHO patients and different PTHLH expression group was analyzed by Chi-square test or Fisher’s Exact Chi-square test. Results are presented as Mean ± standard deviation (SD) from a minimum of three replicates. Difference between groups was evaluated by SPSS 23.0 statistical software Statistical significance was concluded at *P<0.05, **P<0.01, ***P<0.001; P >0.05 represents no statistical significance.

**Figure S1.** Immunohistochemistry. (A-B) Scores indicate PTHLH levels in ECC (DAB staining, Scale bar, 50μm).The scores were calculated by intensity and percentage of stained cells as described in the Supplemental Information.

**Figure S2.** (A) Endogenous PTHLH expression in two ICC cell lines was measured by Western blot analysis. (B-C) Knockdown efficiency of PTHLH in RBE and HCCC-9810 cells by stable transfection of shPTHLH was examined by qPCR and Western blot (*p<0.05). (D) Re-expression efficiency of PTHLH on RBE and HCCC-9810 cells was confirmed by qPCR and Western blot. (E) Western blot results demonstrate that the secreted PTHLH in culture media (PTHLH_CCM_) significantly increased when PTHLH was reintroduced with lentivirus-mediated PTHLH_GFP_.

**Figure S3.** Morphological changes of the cells. Compared with the control, incubation with PTHLH-specific shRNA resulted in elongated morphology cells and less confluent cell growth. When exposed to lentivirus-mediated PTHLH_GFP_, cell growth returned to normal (magnification 100×).

**Figure S4.** (A-B) Soft agar colony formation assay of PTHLH-knockdown and PTHLH-re-expression in 6-well dish (4*10^4^ cells per well) for 3 weeks (n = 3). Representative images (left) and average number of colonies (right) are shown.

**Figure S5.** (A) No significant differences between migration and invasion of HCCC-9810 cells (p>0.05). (B) Re-expression PTHLH facilitated RBE cells migration(p<0.05) not invasion (p>0.05). (C) No significant differences were identified in apoptotic cells of RBE cells by flow cytometry analyses following Annexin V and 7-amino-actinomycin (7-AAD) staining (p>0.05).

**Figure S6.** Endogenous PTHLH re-expression upregulated ATF2 protein expression. (LV-P1,lentivirus-mediated PTHLH_GFP,_ MOI=1; LV-P2, MOI=10; LV-P3, MOI=100)

**Figure S7.**Schematic diagram of the PTHLH proximal promoter. The nucleotide positions and sequences of the putative ATF2 binding site are shown.

**Additional Figure S1.**

**
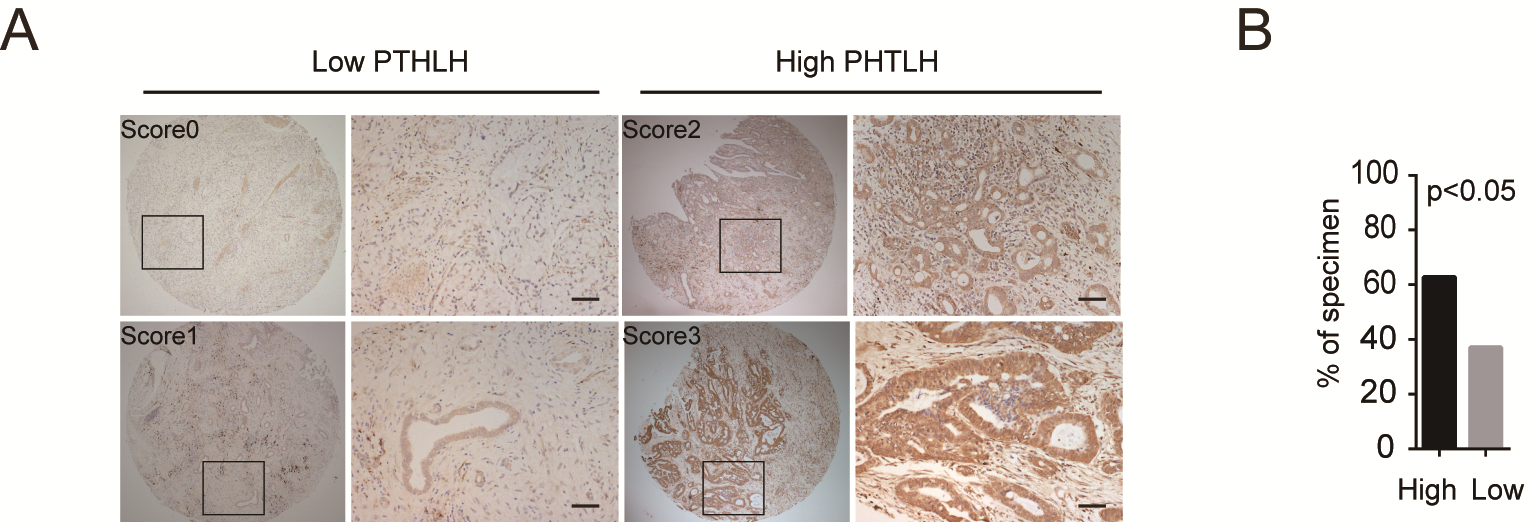
**

**Additional Figure S2.**


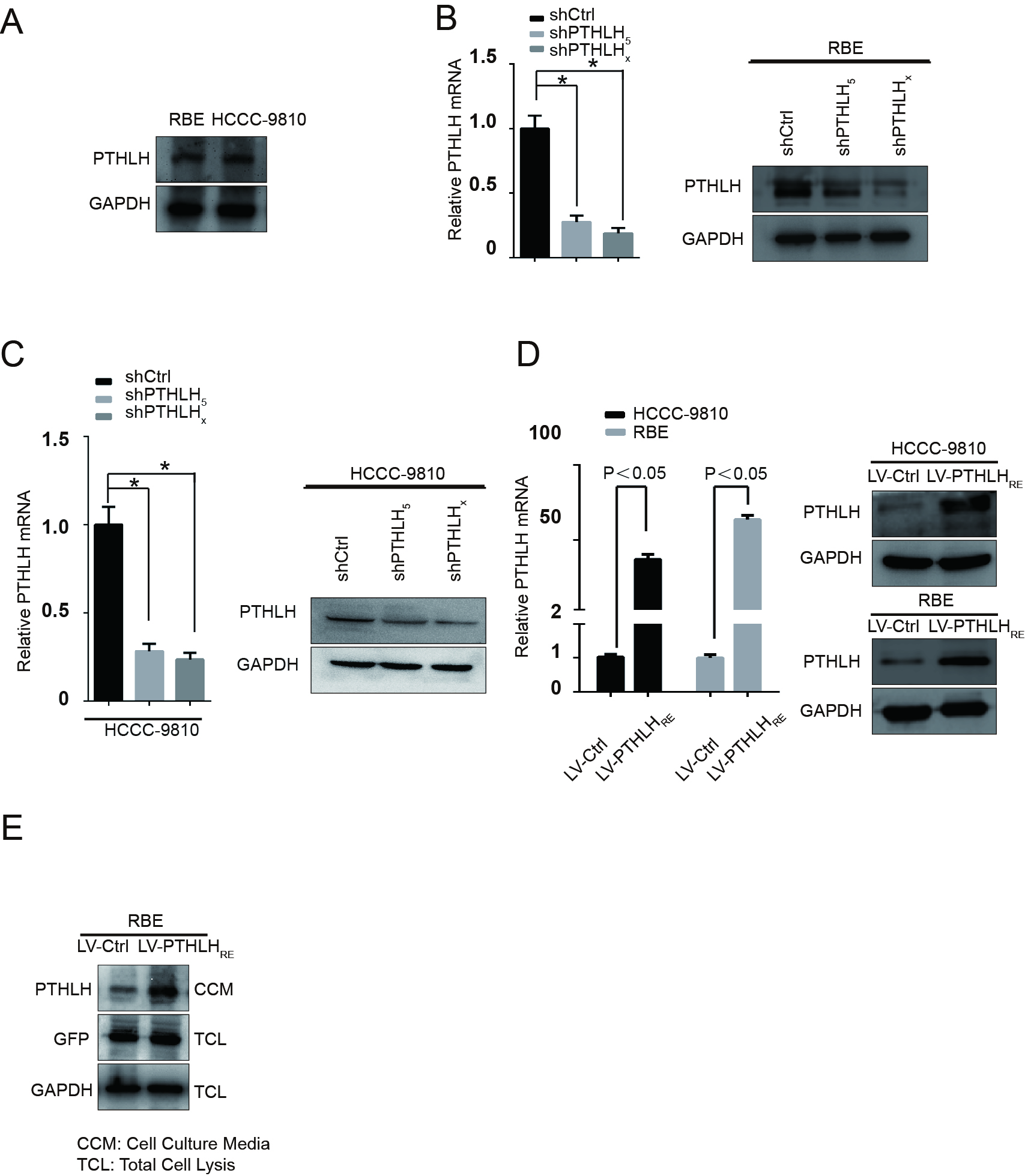


**Additional Figure S3.**

**
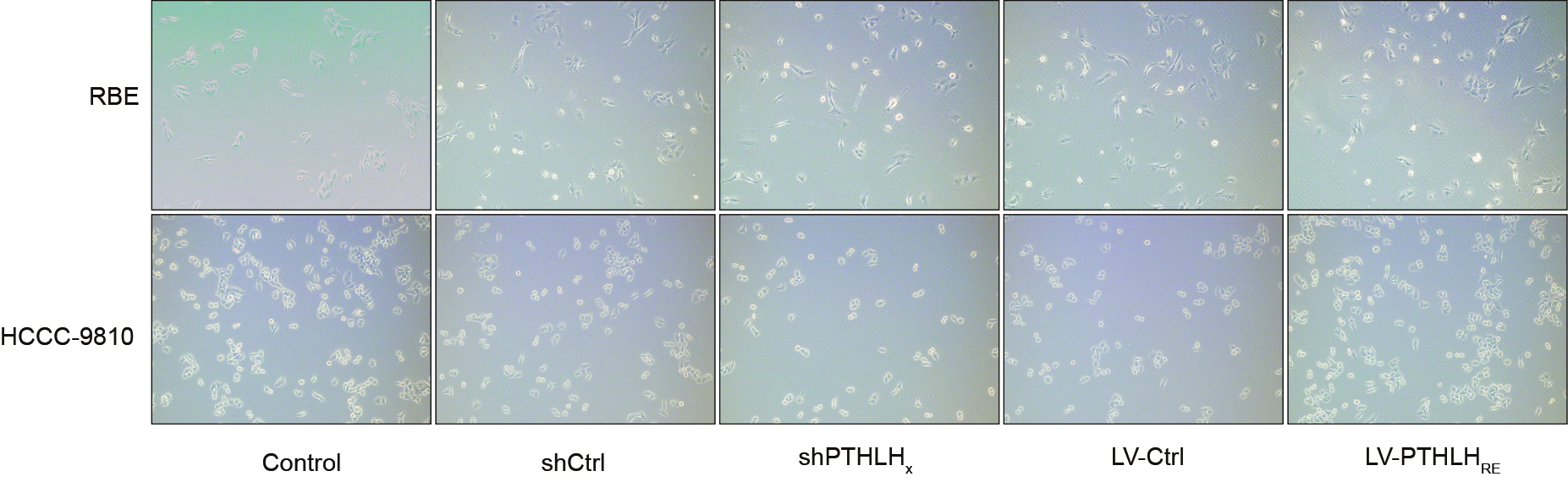
**

**Additional Figure S4.**

**
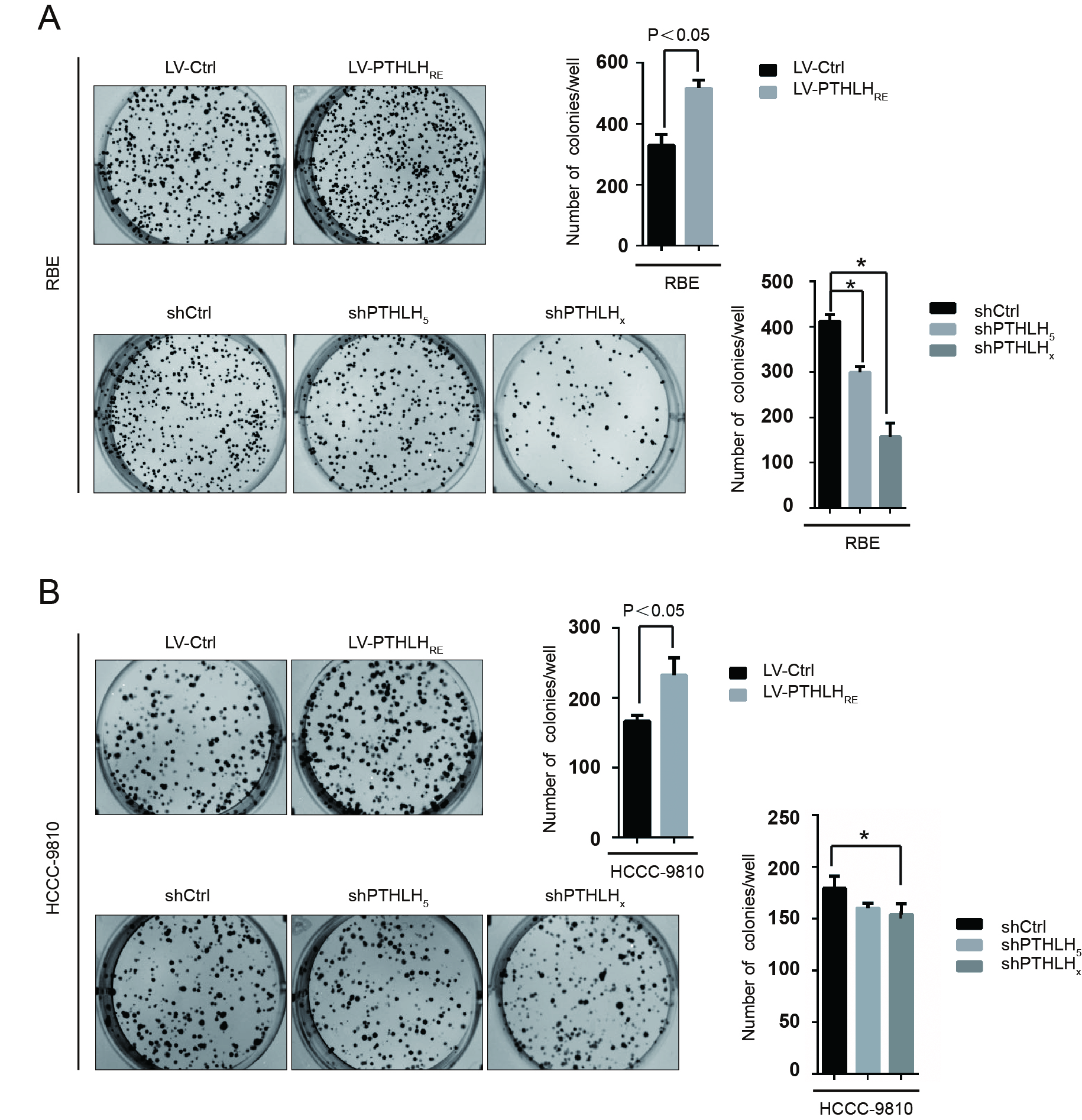
**

**Additional Figure S5.**


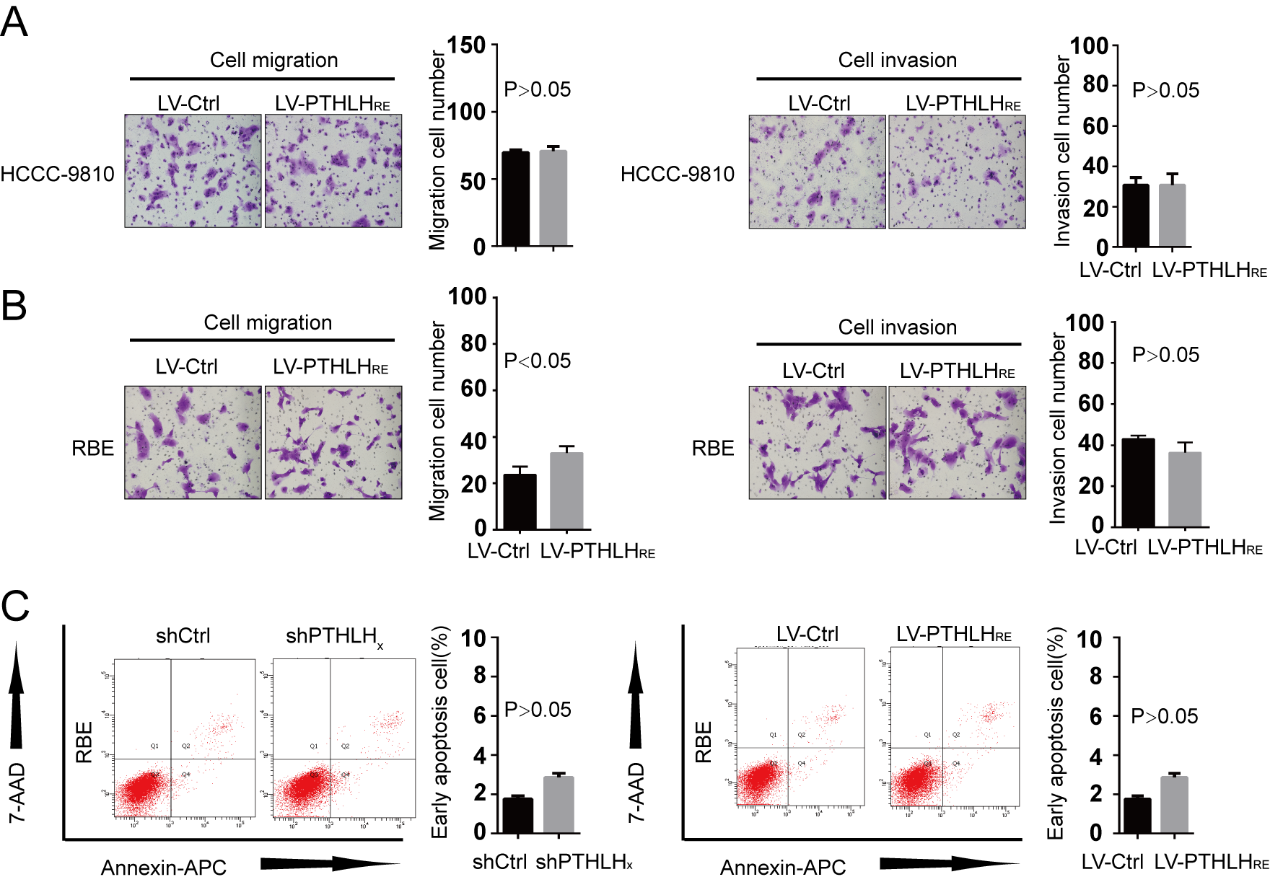


**Additional Figure S6.**

**
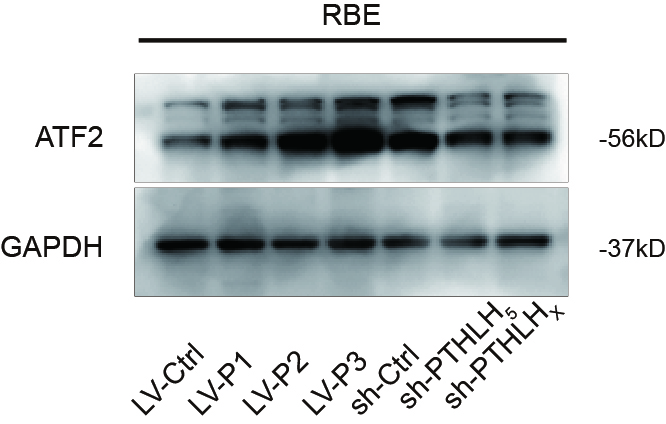
**

**Additional Figure S7.**


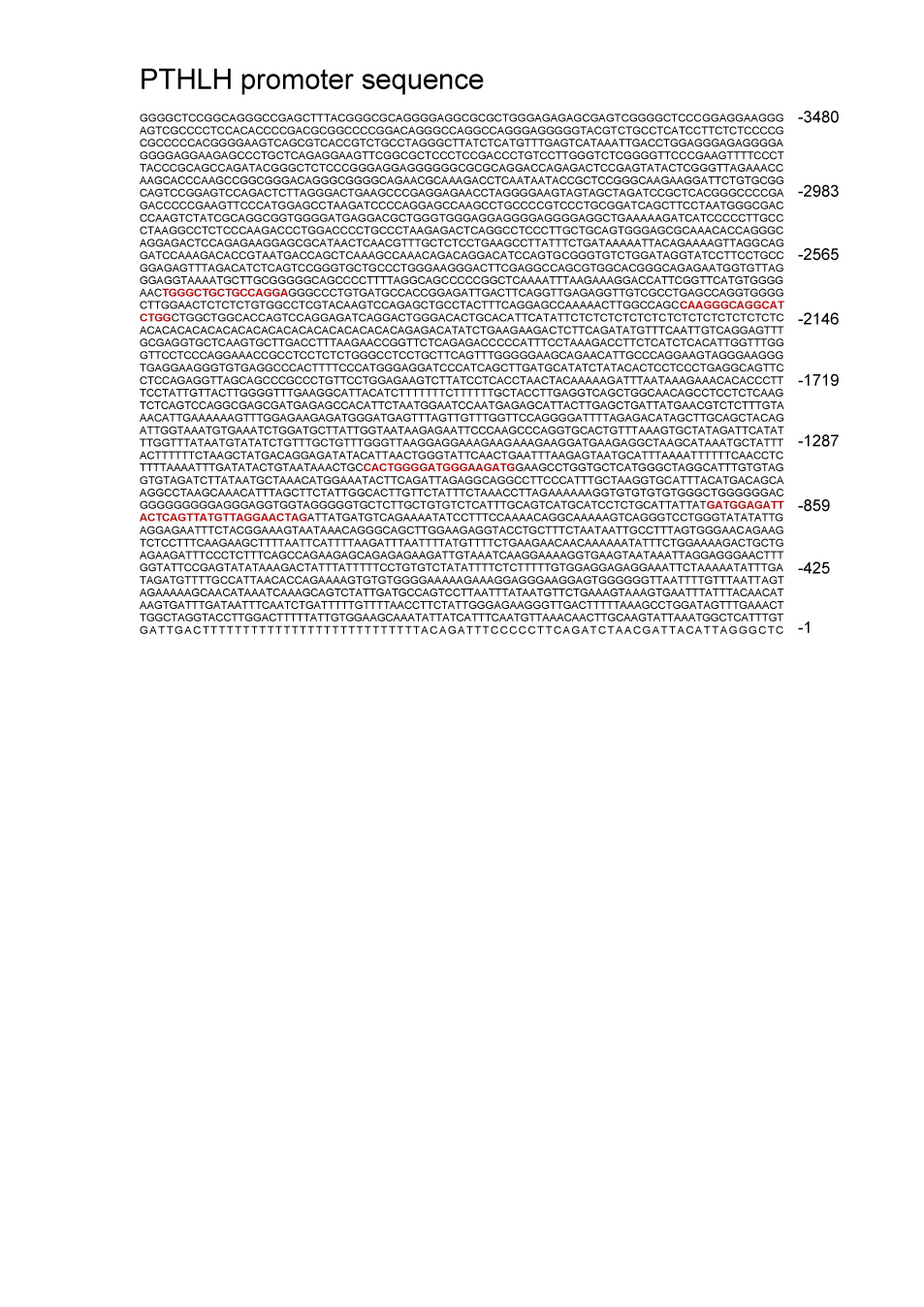


**References**

[1] Lu D, Han C, Wu T, et al. Microsomal prostaglandin E synthase-1 inhibits PTEN and promotes experimental cholangiocarcinogenesis and tumor progression. Gastroenterology. 2011;140(7):2084-2094.

[2] Figueiredo MS, Brownlee GG. Cis-acting elements and transcription factors involved in the promoter activity of the human factor VIII gene. J Biol Chem. 1995; 270(20):11828-11838.
